# Psychological distress and social support among conflict refugees in urban, semi-rural and rural settlements in Uganda: burden and associations

**DOI:** 10.1186/s13031-022-00451-3

**Published:** 2022-05-12

**Authors:** Gloria Seruwagi, Catherine Nakidde, Eric Lugada, Maria Ssematiko, Dunstan P. Ddamulira, Andrew Masaba, Brian Luswata, Eric A. Ochen, Betty Okot, Denis Muhangi, Stephen Lawoko

**Affiliations:** 1 Makerere University School of Public Health, Kampala, Uganda; 2 Department of Social Work and Social Administration, Centre for Health and Social Economic Improvement (CHASE-i), Makerere University, Kampala, Uganda; 3 Agency for Cooperation and Research in Development (ACORD), Kampala, Uganda; 4 Lutheran World Federation (LWF), Kampala, Uganda; 5 Directorate of Governance and Regulation, Ministry of Health, Kampala, Uganda; 6 Department of Public Health Faculty of Medicine, Gulu University, Gulu, Uganda

**Keywords:** Psychological distress, Social support, MHPSS, COVID-19, Urban refugees, Urban / rural, Conflict refugees, Uganda

## Abstract

**Background:**

Recent research shows that psychological distress is on the rise globally as a result of the COVID-19 pandemic and restrictions imposed on populations to manage it. We studied the association between psychological distress and social support among conflict refugees in urban, semi-rural and rural settlements in Uganda during the COVID-19 pandemic.

**Methods:**

Cross-sectional survey data on psychological distress, social support, demographics, socio-economic and behavioral variables was gathered from 1014 adult refugees randomly sampled from urban, semi-rural and rural refugee settlements in Uganda, using two-staged cluster sampling. Data was analyzed in SPSS-version 22, and statistical significance was assumed at *p* < 0.05.

**Results:**

Refugees resident in rural/semi-rural settlements exhibited higher levels of psychological distress [F(2, 1011) = 47.91; *p* < 0.001], higher availability of social interaction [F(2, 1011) = 82.24; *p* < 0.001], lower adequacy of social interaction [F(2, 1011) = 54.11; *p* < 0.001], higher availability of social attachment [F(2, 1011) = 47.95; *p* < 0.001], and lower adequacy of social attachment [F(2, 1011) = 50.54; *p* < 0.001] than peers in urban settlements. Adequacy of social interaction significantly explained variations in psychological distress levels overall and consistently across settlements, after controlling for plausible confounders. Additionally, adequacy of social attachment significantly explained variations in psychological distress levels among refugees in rural settlements, after controlling for plausible confounders.

**Conclusion:**

There is a settlement-inequality (i.e. rural vs. urban) in psychological distress and social support among conflict refugees in Uganda. To address psychological distress, Mental Health and Psychosocial Support Services (MHPSS) should focus on strategies which strengthen the existing social networks among refugees. Variations in social support are a key predictor of distress which should guide tailored need-adapted interventions instead of duplicating similar and generic interventions across diverse refugee settlements.

## Introduction

COVID-19 has massively disrupted the health and wellbeing of population groups worldwide since it was declared a pandemic over a year ago. Beside the fear, anxiety, confusion and frustrations triggered by the pandemic [1], the stringent measures activated by governments to curtail the disease spread may further exacerbate psychological distress [2]. Globally, experts predict that the collective impact of these restrictions on household socioeconomics, health and wellbeing, as well as the social infrastructure of communities will extend beyond the lifespan of the pandemic [1, 3–8]. Cross-sectional studies showing high burden of intimate partner violence, and chronic conditions such as depression and anxiety in different settings during the COVID-19 era are early signals of this prognosis [9–13].

Despite the growing body of evidence suggesting deteriorating psychosocial wellbeing at population level, equivocal data from refugee populations is lacking. Currently surpassing 80 million in number globally, conflict refugees are particularly at heightened risk of psychological distress when contrasted with host communities. A myriad of psychosocial problems including Post-Traumatic Stress Disorder (PTSD), depression and anxiety [14, 15], and psychological distress triggered by fears of apprehension, risk of deportation, difficult living conditions, poor access to health, social, communication, financial and legal services [16–22], have been reported in refugee settings. This is in addition to the stressors imposed by restrictions to contain COVID-19 pandemic at the population level [1, 3–13]. An assessment of psychological distress status and psychosocial support needs for refugees is therefore particularly imperative in the pandemic era. A number of factors have been associated with mental and psychosocial health of refugees including demographic and behavioural indicators such as gender, marital status, nationality, alcohol and substances abuse [23, 24]. However, the role of social support, a potential modifiable risk factor, has not received equivocal attention in the humanitarian population.

Yet, social support can play an important role in preventing, containing or moderating the psychological wellbeing of populations through several mechanisms. While some scholars have emphasized its role as a stress buffer [25, 26], others have highlighted its protective function as a coping facilitator [27, 28] as well as its role in health promotion [29]. As a stress buffer, social support is envisioned to alleviate the detrimental impact of stressful life events by modifying negative appraisals and promoting problem solving strategies [28, 30, 31]. Some scholars argue however that during times of severe or chronic distress, the buffering effect of support may be limited [32, 33]. In contrast, as a coping facilitator, social support is envisaged to provide regular directly rewarding experiences such as positive affection, which prevent the development of psychologically distressful outcomes [26, 31]. Moreover, a supportive network is hypothesized to promote behaviors beneficial to health such as timely seeking of healthcare and adaptation of healthy lifestyles (e.g. healthy nutritional choices and physical activity) [25, 31, 32], thereby reducing the likelihood of psychological distress.

Although population studies have alluded to a breakdown in social support networks during the COVID-19 pandemic [1, 4], there is a dearth of studies investigating the availability and adequacy of such networks, and their association with psychological distress particularly in refugee populations where the need is augmented. Moreover, refugees live under varying conditions with some residing in urban and others in rural settings, and this has implications on vulnerability to social and health problems. For instance, while living in urban settings may increase access to a social network (e.g. through gainful employment), it is also associated with psychological stressors that accompany increased population density and diversity such as unemployment, violence, marginalization and exposure to health-risk behaviours [32–34]. Additionally in some countries (e.g. Uganda the context of this work), the settlement of refugees in rural/urban areas has been characterised by cultural homogeneity [35] i.e. refugees in each settlement are predominantly of the same nationality, whose role in strengthening social integration among the refugees remains elusive. While on the one hand the collectivist orientation of such settlements may promote social interaction by virtue of shared cultural norms and interest, efforts to integrate within the host culture on the other hand may disrupt such intentions [34, 36, 37]. Thus, contrasting hypotheses on the relationship between distress, social support and urbanization in refugee populations warrant investigation on their own right.

Using data from Uganda, we assessed for:

1. Differences in levels of psychological distress between refugees in urban, semi-rural and rural settlements.
2. Differences in levels of perceived social support between refugees in urban, semi-rural and rural settlements.
3. Differences in associations between psychological distress and social support between refugees in urban, semi-rural and rural settlements.

## Methods

### Study context

Uganda is host to nearly 1.5million conflict refugees from neighbouring countries of Rwanda, Burundi, South Sudan and Democratic Republic of Congo [38]. These refugees exhibit poor living conditions, pre-existing mental and psychosocial challenges [38–40]. The inaction caused by COVID-19 preventive measures, starting from the first case identification on 21 March 2020, including restrictions on mass gatherings, public transport, entry and exit at border points, and lockdown on several social services [40] is envisioned to have impacted further on psychological distress in this already vulnerable population. Indeed, emerging data in vulnerable groups of refugee women and slum-dwellers suggest that COVID-19 has exacerbated their risk for stigma, all forms of violence and financial disadvantage [41, 42].While researchers have predicted the economic, psychosocial, physical, and other consequences of COVID-19 on refugees/migrants in Uganda and beyond based on previous epidemics [43–46], there is a dearth of evidence on the burden of psychological distress, social support and strength/nature of associations between these phenomena in refugee settings during the COVID-19 pandemic. The current work intends to fill this gap in the evidence using Uganda as a case study. Such data could be useful in the design of interventions to cushion psychosocial problems among refugees through the modification of social support agents such as availability and adequacy.

### Study site and population

We conducted the research at 3 large refugee settlements in different regions of Uganda, hosting over 400,000 refugees:

i. Kisenyi, refugee settlement, an urban refugee setting in the centre of the capital city (Kampala) hosting over 70,000 refugees of mainly Somali origin. The refugees live integrated with their host.
ii. Kyaka II refugee Settlement in the Southwestern part of Uganda, a semi-rural refugee setting hosting multinational refugees from the Democratic Republic of Congo (DRC), Burundi and Rwanda totaling approximately 124,000 refugees. The refugees live partly segregated from their host but with freedom of movement and shared services. The region can be considered as semi-rural, with a blend of rural and urban activities (e.g. farming and industrial activities)
iii. Adjumani refugee settlement in North-West Nile Uganda, hosting about 214,000 refugees predominantly of South Sudanese nationality. The refugees live rather segregated from their host but with freedom of movement and shared services. The region is considered as rural, with agriculture as the main activity.

### Study design

Cross-sectional survey data on various health and social indicators was gathered from 1014 refugees randomly selected from each of the study sites. For the current study, data on psychological distress, social support, demographic, social and behavioral indicators was of primary interest.

### Sampling procedure

Participants were sampled using a two-staged cluster sampling procedure in each settlement. The first stage involved selecting clusters of zones in the main settlement using systematic random sampling with probability proportional to zone size (PPS). The second stage constituted systematic random sampling of households in selected zones. For instance, a Research Assistant picked a household at random within the zone, and then decided to visit every nth household (e.g. 3rd) moving in a specific direction (e.g. eastward) until his/her required quota (e.g. 50 households) was completed. Random numbers procedures were used to choose one adult household member (i.e. 15 years and above) from among all adults in the household to constitute the final participant. This procedure resulted in 1014 refugees, with the following distribution among the settlements: Adjumani n = 342; Kyaka 354; Kisenyi n = 318.

### Ethical considerations

Thirty Research Assistants (RAs) were trained to collect data using mobile tablets, in a bid to reduce inter-individual contact and the risk of COVID-19 spread during interviews. The training oriented RAs on the purpose of the study; ethical considerations; data collection methods and tools; COVID-19 prevention, symptoms, measures and precautions; and standard operating procedures (SOPs) in fieldwork in light of COVID-19. The training also involved testing of the data collection tool among a purposively selected refugee sample of n = 30 in each of the 3 settlements, from zones neighboring but not included in the main study. Slight adjustments were made to data collection tools following this exercise.

Informed consent was received from all participants, and confidentiality was considered by inquiring of participants whether they felt safe to partake in study, emphasizing that participation was voluntary, and giving the participant liberty to choose whether he/she preferred another time and/or venue for the interview. The potential risk and benefits of the study were explained to all participants and in light of the heightened risk of COVID-19 transmission, we developed Standard Operational Procedures (SOPs) for protection of refugees as well as data collectors, guided by Safety and Security Strategy for COVID-19 of the World Health Organization (WHO) and Uganda Ministry of Health COVID-19 guidelines.

The study was approved by the Makerere University School of Public Health Institutional Review Board (MakSPH IRB) and the Uganda National Council of Science and Technology (UNCST), the two bodies governing academic research in Uganda. Additionally, the Ministry of Health (MoH), Kampala Capital City Authority (KCCA) and the Office of the Prime Minister (OPM), which is in charge of refugee affairs, gave clearance for execution of the study.

### Data collection tools and study variables

A comprehensive questionnaire covering several areas of relevance to public health and COVID-19 was developed. For the current study, the following variables were of interest.

#### Dependent variables

The dependent variable for the study was psychological distress, measured using Kessler’s Psychological Distress Scale (K-10) [47], a 10-item instrument measuring distress in terms of feelings of nervousness, hopelessness, tiredness, restlessness, fidgety, depressed mood, sadness, worthlessness, cheerlessness and loss of effort, during the past 14 days, with a 5-level response ranging from none of the time (score 1) to all of the time (score 5). A composite score for psychological distress is calculated for each participant as the sum of responses to the the 10 items. Thus, individual scores for psychological distress scale ranged from 10 to 50, with higher scores indicative of higher psychological distress. Cronbach’s alpha testing for internal consistency/reliability of K-10-Scale for the current sample was 0.91 indicating very high reliability.

#### Independent variables

The main independent variable for this study was social support, with the aim to assess its association with psychological distress, and whether such associations differ between refugees in rural, semi-rural and urban settlements. Social support was measured using a modified version of the Interview Schedule for Social Integration (ISSI) [48], which assesses social support in terms of the *Availability and Adequacy of Social Interaction and Social Attachment.*

*Availability of Social Interaction* (AVSI) was assessed using six items inquiring of participants to indicate if they have anyone/persons: with whom they have common interest, meet and talk to regularly, can speak with openly, can borrow things from and can turn to when in trouble. This was coded as 1 if the answer was in affirmative and zero if the response was”No”. A composite score was formed ranging between 0 and 6 to represent, with higher scores indicative of higher availability. The participants were in addition requested to rate the *Adequacy* of these person/persons by inquiring if they desired more (coded as 1), less (coded as 1) or no change (coded as zero). Desiring “more” or “less” in a specific item was considered “inadequate”, while desiring neither more nor less was considered “adequate”. Thus, *Adequacy of Social Interaction* (ADSI) was rated on a total scale ranging between 0 and 6, with higher scores indicative of lower adequacy. Cronbach’s alpha testing for internal consistency/reliability of Availability and Adequacy of Social Interaction respectively for the current sample was 0.71 and 0.81 respectively, indicative of good reliability.

*Availability of Social Attachment* (AVSA) was assessed based on six items inquiring of participants to indicate using a “Yes” (coded as 1) or “No” (coded as 0) response regarding whether there is someone special: from whom they derive support, they feel close to, they share happy moments, they can embrace for comfort, who appreciates what they do, and with whom they can share inner thoughts. For social attachment, composite individual scores are calculated as the sum of responses to each item. Thus, scores for social attachment range between 0–6, with higher scores representing higher availability. *Adequacy of Social Attachment* (ADSA) was assessed by inquiries to participants on whether they desired more (coded 1), less (coded 1) or no change (coded 0) regarding the mentioned attachments. Thus, scores for Adequacy of Social Attachment ranged between 0 and 6, with higher scores indicative of lower adequacy. Cronbach’s alpha testing for internal consistency/reliability of Availability and Adequacy of social attachment respectively for the current sample was 0.55 and 0.87 respectively, indicating low and high reliability respectively.

Other independent variables included in the study were:

*Demographic* and *Social* characteristics i..e. refugee settlement (Rural, Semi-rural, Urban), nationality (South Sudanese, Congolese, Somali, Rwandese, Burundian), gender, age, marital status, religion, income (earnings per week), employment status and education (highest level achieved).

*Behavioral characteristics* assessed by indicators including: alcohol use which were assessed by asking participants if they took alcohol regularly (with “Yes/No” response), smoking assessed by inquiring of participants if they currently smoke (with “Yes/No” response)and physical activity assessed by inquiring of participants how often they engaged in exercise in a week (with response options “never”, “once”, “2–3 times” and “4 or more times”). As these variables are from previous studies generally known to be associated both with social support and psychological distress, it is prudent to adjust for them in the main analyses to control for possible confounding.

COVID-19 symptoms were assessed by asking participants if they had currently or within the past 14 days experienced/exhibited symptoms of coughing, sneezing, running nose, sore throat, difficulty in breathing, loss of taste, and loss of smell. The number of symptoms was calculated per individual and used in the analyses to represent COVID-19 risk. This variable therefore ranged from 0 to 8, with higher scores indicative of higher risk of COVID-19 transmission.

Detailed categorization of all variables are shown in Table 1.

**Table 1:** Demographic, social, behavioural and clinical characteristics of participants

Characteristic	n	%^a^
Refugee settlement		
Adjumani (Rural)	342	33.8
Kyaka II (Semi-rural)	354	35.0
Kisenyi (Urban)	317	31.2
Nationality		
South Sudanese (100% of Rural)	343	33.9
Congolese (94% of Semi-rural)	342	33.8
Somali (95% of Urban)	308	29.8
Others (Rwandese, Burundians) (80% in Semi-rural)	26	2.5
Age		
15–24	220	21.7
25–34	355	35.0
35–44	254	25.1
45–54	84	8.3
55–64	67	6.6
65–74	25	2.5
75–84	8	0.8
Gender		
Male	318	31.4
Female	693	68.4
Religion		
Moslem	307	30.3
Catholic	188	18.6
Protestant	370	36.5
Other (e.g. Adventist, Jehovah’s Witness)	14,314.6	
Occupation		
Employed	34	3.4
Self-employed	120	11.9
Unemployed	712	70.3
Student	113	11.2
Other (shifting e.g. vendor, retailer etc.)	32	3.1
Earnings per week (Ugandan Shillings, UGX)		
Less than 50,000 UGX	577	57.1
50,000–100,000 UGX	87	8.7
100,000–200,000 UGX	35	3.5
Over 200,000 UGX	33	3.3
Highest education level		
No education	407	40.2
Primary level	303	29.9
Secondary level	230	22.7
Tertiary or vocational	24	2.4
University	46	4.5
Smoker		
Yes	38	3.8
No	970	95.8
Drink alcohol		
Yes	72	7.2
No	931	91.9
Exercise		
Never	450	44.4
Once	175	17.3
2–3 times	258	24.5
4 times or more	127	12.5
Symptoms of COVID-19		
Cough	43	4.2
Sneezing	39	3.8
Running nose	28	2.8
Sore throat	20	2.0
Difficulty breathing	20	2.0
Bodily pain	154	15.3
Loss of sense of smell	22	2.2
Loss of sense of taste	27	2.6
At least one of symptoms above	223	22.3

^a^Percentages may not add up to 100% due to missing values for some variables. For COVID-19, percentages add up to more than 100% due to possibility of participants presenting with multiple symptoms

### Statistical analysis

Cronbach’s Alpha coefficients were calculated to assess for reliability (internal consistency) of the dependent variable and the main independent variables i.e. Kessler’s Psychological Distress Scale and ISSI sub-scales) in the current sample. To compare the burden of psychological distress between rural, semi-rural and urban refugee populations, Analysis of Variance (ANOVA) was used, and post hoc tests with Bonferroni correction applied to account for multiple pairwise comparisons of means between the three sub-populations. Similarly, to examine differences in availability and adequacy of social support between refugees in rural, semi-rural and urban settlements (ANOVA) were used, with post hoc tests according to Bonferroni method applied. To assess for bivariate associations between psychological distress and sex, independent sample t-tests was used. To assess for bivariate associations between psychological distress and settlement, nationality, occupation and religion ANOVA (contrasting 3 or more means) was used respectively, with post hoc corrections according to Bonferroni method. To assess for bivariate associations between psychological distress and age, education, smoking, alcohol use, exercise and social support indicators respectively, Pearson’s Correlations tests was used respectively.

To assess the independent association between psychological distress and social support while controlling potential confounder, all independent variables exhibiting statistical significance in the bivariate tests were entered in Multivariable Linear Regressions (MLR). Assumptions for Multiple Linear Regression (MLR) i.e. linearity, homoscedasticity of variance and multicollinearity were met. Deviations were noted regarding the normality assumption though a preference not to transform the data was adopted due to several reasons as discussed under study limitations.

Ordinal and continuous independent variables that were significant in the bivariate analyses were entered in the regression in their original form, while nominal variables were transformed to dummy variables prior to entry in the regression. Regressions were run for the entire sample to assess associations between psychological distress and social support among refugees in general, while controlling for potential confounders. Additionally, regressions analyses stratified by settlement were run to compare the association between psychological distress and social support between settlements (i.e. rural, semi-rural and urban) while controlling for potential confounders. The same variables entered in the un-stratified analyses were included in the stratified analyses except for settlement (the stratification variable), which was excluded in the stratified analyses.

SPSS version 22 was used for all analyses and a statistical significance of *p* < 0.05 assumed for all tests.

## Results

### Demographic, social, behavioural and clinical characteristics of participants

Study participants were equally distributed across the rural, semi-rural and urban settlements of Adjumani, Kyaka IIand Kisenyi respectively (Table 1). Majority of participants were of South-Sudanese, Congolese or Somali origin, with a specific nationality predominating in each settlement (i.e. the urban settlement of Kisenyi was dominated by Somalis 95%, rural settlement of Adjumani dominated by South Sudanese (100%), and semi rural settlement of Kyaka II dominated by Congolese (94%).). Majority of the refugees were: aged under 45 years (over 80%), of female sex (65%), and Protestants (36%). Many refugees were unemployed, in the low-income bracket, and were uneducated. Regarding behavioral characteristics, few participants were smokers, or drank alcohol regularly though many (44%) were physically inactive by way of exercise. Twenty two percent (22%) reported having at least one symptom of COVID-19, with bodily pains being the most prominent symptom (15%) (Table 1).

### Participants ratings of availability of social interaction (AVSI) and adequacy of social interaction (ADSI) by settlement

As exhibited by the Confidence Intervals for the mean of AVSI, refugees rated their AVSI on average between 4.6 and 4.8, on a scale ranging between 0 and 6 (the higher the score the higher the availability) (Table 2). Differences were observed between settlements [F(2, 1011) = 82.24; *p* < 0.001] and confirmed by post-hoc test using Bonferroni correction.

**Table 2:** Refugees ratings of levels of social support and psychological distress: overall and by settlement

Variable (Range)	Rural Mean (CI)	Semi-rural Mean (CI)	Urban Mean (CI)	Total Mean (CI)
AVSI*** (0–6)	5.1 (4.9–5.2)	5.1 (4.9–5.2)	3.7 (3.5–3.9)	4.6 (4.5–4.7)
ADSI*** (0–6)	4.3 (4.1–4.5)	4.6 (4.4–4.8)	3.1 (2.9–3.4)	4.0 (3.9–4.2)
AVSA*** (0–6)	5.3 (5.0–5.5)	5.7 (5.5–5.9)	4.9 (4.8–5.1)	5.3 (5.2–5.4)
ADSA*** (0—6)	4.7 (4.5–4.8)	4.6 (4.4–4.8)	3.2 (2.9–3.5)	4.2 (4.1–4.3)
Psychological Distress*** (10–50)	21.2 (20.5–22.4)	23.7 (22.7–24.8)	17.3 (16.5–18.0)	20.9 (20.4–21.5)

***ANOVA f-test statistically significant at *p* < 0.001

As exhibited by the Confidence Intervals for the mean of ADSI on the other hand, refugees rated ADSI on average between 3.9 and 4.2, on a scale of range 0–6 (higher scores denote lower adequacy). Differences were observed between settlements [F(2, 1011) = 54.11; *p* < 0.001], and confirmed by post-hoc test using Bonferroni correction.

### Refugees ratings of availability of social attachment (AVSA) and adequacy of social attachment (ADSA) overall and by settlement

As exhibited by the Confidence Intervals for the mean of AVSA on average, refugees rated their AVSA rather high (i.e. between 5.2 and 5.4 on a scale of range 0–6 (Table 2). Differences were observed in AVSA ratings between settlements [F(2, 1011) = 47.95; *p* < 0.001], and confirmed by post-hoc test using Bonferroni correction..

As exhibited by the Confidence Intervals for the mean of ADSA on the other hand, refugees rated adequacy of social attachment (ADSA) on average between 4.1 and 4.3 on a scale ranging between 0 and 6. (Table 2). Differences were observed in ratings between settlements [F(2, 1011) = 50.54; *p* < 0.001], and confirmed by post-hoc test using Bonferroni correction.

### Refugees ratings of psychological distress overall and by settlement

According to Table 2, levels of psychological distress ranged on average between 20.4 and 21.5 across settlements on a scale ranging from 10–50, where higher scores denote higher psychological distress. Differences were observed between settlements [F(2, 1011) = 47.91; *p* < 0.001], with urban settlements exhibiting lower distress on average, and confirmed by post-hoc test using Bonferroni correction.

### Association between psychological distress and demographic, social, behavioural and clinical indicators

Psychological distress levels varied significantly according to demographic, social, behavioural and clinical characteristics of refugees (Table 3). Distress levels varied by nationality [F(3, 1010) = 35.3; *p* < 0.001] (Table 3), sex [t(1010) = 1.95; *p* = 0.05], religion [F(3, 1010) = 28.3; *p* < 0.001], and employment status [F(4, 1009) = 7.0; *p* < 0.001]. In addition, Pearson’s Correlation Coefficients (r) showed that levels of psychological distress increased with increasing age [r = 0.18; *p* < 0.01], reduced with increasing level of education [r = − 0.20; *p* < 0.001], reduced with increasing level of physical activity [r = − 0.25; *p* < 0.001] and increased with increasing number of COVID-19 symptoms [r = 0.16; *p* < 0.001] among the refugees.

**Table 3:** Bivariate association between demographic, socio-behavioural and social support indicators and psychological distress

Characteristic	Mean (SE)
Nationality	
South Sudanese	21.4 (0.5)
Congolese	23.7 (0.5)
Somali	16.9 (0.3)
Other (i.e. Rwandese, Burundians)	23.6 (1.8)
Sex*	
Female	21.3 (0.3)
Male	20.1 (0.5)
Religion	
Moslem	16.9 (0.4)
Catholic	22.4 (0.7)
Protestant	23.3 (0.5)
Other (e.g. Adventist, Jehovah Witness)	21.1 (0.7)
Occupation	
Formal employed	17.5 (1.3)
Self-employed	18.7 (0.6)
Unemployed	21.9 (0.3)
Student	18.7 (0.8)
Other (shifting e.g. vendor, retailer etc.)	19.8 (1.3)

	Pearsons Correlation Coefficient (r)
Age (years)*	0.18
Earnings per week (Shillings)	− 0.02
Highest Education Level*	− 0.2
Smoking (Yes/No)	− 0.04
Drink Alcohol (Yes/No)	− 0.02
Exercise (Frequency)*	− 0.25
Availability of Social Interaction	0.03
Adequacy of Social Interaction*	− 0.18
Availability of Social Attachment	− 0.06
Adequacy of Social Attachment**	− 0.18
Number of COVID-19 Symptoms	0.16

*T-test for difference in means or Correlation Coefficients (r) significant at *p* < 0.05; **ANOVA F-tests or Correlation Coefficients (r) statistically significant at *p* < 0.001

### Bivariate association between social interaction, social attachment and psychological distress

Significant correlations were observed between Psychological distress and social support indicators (Table 3). ADSI exhibited a negative correlation with psychological distress (i.e. as ADSI scores increased on average, psychological distress scores reduced) [r = − 0.18; *p* < 0.01]. Similarly, as ADSA scores increased on average, distress scores reduced [r = − 0.18; *p* < 0.01]. Thus, with increasing ADSI and ADSA, psychological distress decreased.

### Multiple linear regressions assessing association between psychological distress and social support, adjusted for demographic, social, behavioural and clinical factors among refugees in general and stratified by settlement

As indicated in Table 4 by the standardized regression coefficients (Beta), ADSI remained significantly associated with psychological distress even after controlling for plausible demographic, social and behavioural confounders, overall and by settlement, i.e. With increasing ADSA, Psychological Distress reduced. ADSI on the other hand lost statistical significance when plausible confounding was adjusted for in the analysis of all refugees, but remained significantly associated with distress in the analysis among refugees in rural settlements (i.e. with increasing AVSA, distress levels reduced on average).

**Table 4:** Multiple linear regression: adjusted associations between psychological distress and and social support indicators, overall and stratified by settlement, and COVID-19 symptoms stratified by settlement

Characteristic	Overall	Urban Standardized Beta	Semi-rural Standardized Beta	Rural Standardized Beta
Adequacy of Social Interaction (ADSI)	− 0.11**	− 0.18**	− 0.18**	− 0.18**
Adequacy of Social Attachment (ADSA)	− 0.08	− 0.09	0.07	− 0.29***

*p* < 0.05; **p < 0.01; ***p < 0.001. Adjusted for sex, religion, occupation, age, frequency of exercise and COVID symptoms. Additionally adjusted for settlement in overall analysis

### Multiple linear regressions assessing the association between psychological distress and social support, adjusting for demographic, social and behavioural factors, and COVID-19 symptoms stratified by refugee settlement

As shown in Table 4 by the standardized regression coefficients (Beta), ADSI remained statistically significantly related with psychological distress in urban, semi-rural and rural settlements respectively, even after controlling for demographic, social, behavioural and clinical indicators. In addition, with increasing ADSI levels of psychological distress decreased among refugees in rural but not semi-rural or urban settlements.

## Discussion

Few studies have scrutinised the individual level social resources available to refugees and how these may manifest on their psychological wellbeing. Accordingly, the primary objective of the current study was to assess the burden of psychological distress and analyze its relationship with social support among refugees resident in urban, semi-rural and rural settlements in Uganda, while controlling for plausible confounding with demographic, social, behavioral and clinical (i.e. COVID-19) characteristics of the refugees.

### Burden of psychological distress in urban, semi-rural and rural refugee settlements

Levels of psychological distress were on average moderate among refugees, but with notable variations across settlements, i.e. significantly higher distress levels were observed among refugees resident in semi-rural and rural settings when contrasted with peers resident in urban settlements. While such data, to the best of our knowledge is previously lacking in refugee cohorts, population studies have generated contradictory results in this respect. Some scholars have supported the notion of heightened psychological distress in urban areas due to stressors related with urbanization such as unemployment, violence, marginalization, discrimination, and increased exposure to health-risk behaviours, while other researchers have envisioned residents in urban settings to have an upper edge in health because of better access to health and social services [33–35]. Our findings could reflect the latter circumstances.

### Availability and adequacy of social interaction and social attachment overall and in urban, semi-rural and rural refugee settlements

Refugees rated their availability of social interaction (AVSI, which encompassed access to resources required for regular social activities such as conversation and meeting people) and availability of social attachment (AVSA, which covered access to social relations of emotional relevance such as embracing for comfort and sharing inner thoughts) as high, but rated adequacy of such resources (i.e. whether such resources were perceived sufficient) relatively lower in general. In addition, while AVSI and AVSA were higher rated among refugees residing in rural/semi-rural settlements when contrasted with peers residing in urban settlements, Adequacy of Social Interaction (ADSI) and Availability of Social Attachment (ADSA) were in general rated higher among refugees in Urban settlements than peers in rural/semi-rural settings. These mixed findings are difficult to reconcile. Given that urban refugees in Uganda live in integrated rather than separate settlements with the host communities unlike rural peers, it could be postulated that they have more opportunities for social networking with host community members, congruent with previous works in the general populations linking better access to support networks to urban settlement [33–35]. Our results with regard to AVSI and AVSA may be a reflection of such circumstances. On the other hand, living in gazetted areas for refugees of homogenous nationality, as is the case in Uganda’s rural and semi-rural refugee settlements, may enhance accessibility to support networks by virtue of shared norms, traditions and values [34, 36, 37]. Our results regarding ADSI and ADSA could be a reflection of this notion. Variations in findings regarding social support could also be explained by the methodology differences. Social support is a multi-dimensional concept and there are significant variations in its conceptualization between studies. For instance, some studies have measured social support in terms of the form of support received (e.g. emotional support, instrumental support, cognitive guidance, informative and appraisal support) [27, 49], while others have focussed on the morphology of the network (e.g. size) [50, 51], or value of relationships within the network (e.g. relationship reciprocity) [25, 52–55]. Our study utilized a tool which incorporates a cocktail of these dimensions. Caution should therefore be taken when comparing studies in the field.

### Association between psychological distress and social support among refugees in general, and refugees in urban, semi-rural and rural settlements

Following the various analyses (bivariate and multi-variate), it is concluded that AVSI and AVSA may not be independently associated with distress levels overall and by settlement. On the other hand, ADSI and ADSA proved to be significantly associated with psychological distress. In addition ADSA proved to be significant only among refugees in rural settings. Two conclusions can be drawn from these results: firstly, that it is the adequacy rather than the availability of the social network that may lead to psychological distress in refugee populations; and secondly, that the association between psychological distress and ADSA may be sensitive to rural vs urban settlement. These findings largely corroborate previous research in general population studies [50, 51], and contribute new data to the refugee literature demonstrating that variations in the levels of psychological distress among refugees is to a larger extent dependent on the perceived meaning and value of the support network (here measured by adequacy), than the support network’s morphological aspects (here measured in terms of availability). The lack of significance of AVSA and AVSI however should underscore the need to investigate other pathways through which support is beneficial for psychological distress control, as our design allowed only for assessment of associations in general. As previously mentioned in the introduction, scholars have distinguished between different pathways through which social support is associated with health outcomes including the direct impact [31], stress buffer [25, 26] and coping facilitator hypotheses [27, 28]. Our study design does not allow for the separate analyses of these mechanisms.

### Implications and recommendations

The burden of psychological distress and social support in refugee settings emphasize the need for surveillance of these phenomena as a basis for informed action. The need to revitalize, strengthen or re-structure existing social networks cannot be overemphasised, with observance of COVID-19 prevention measures notwithstanding. Humanitarian actors will need to invest more in mental health and psychosocial support services (MHPSS), tailoring these interventions to the unique needs of different refugee categories by settlement. Unnecessary duplication of intervention could be avoided with this strategy. Strategies to support and strengthen the existing social relations at disposal to refugees (i.e. family and current friends), in preference to increasing availability of social networks (e.g. increasing number of social relations), should be the goal of MHPSS if levels of psychological distress at group level are to be effectively controlled among refugees.

### Study limitations

The study’s cross-sectional design does not permit casual inference. While we can ascertain an association between distress and social support, we can only speculate on the mechanism of such associations. Secondly, the homogenous cultural composition of refugees in each of the studied settlements (i.e. predominance of Somalis in the urban settlement vs. South Sudanese and Congolese in the rural and semi-rural settlements respectively) makes it difficult to disentangle associations between distress and urban/rural settlement on the one hand from associations between distress and country of origin on the other hand. Thus, the observed differences in distress between settlements could be masking differences in distress due to nationality. An attempt to rectify this by way of analysis, however, would lead to multicollinearity. Thus, we could adjust for it in our study. Thirdly, we used the K-10 and ISSS as measures of Psychological Distress and Social support respectively in our study, although these measures are not previously validated in Uganda or similar contexts to the best of our knowledge. We cannot rule out the plausibility of contextual inadequacy of the instruments. To  mitigate this risk however, we piloted the instruments in smaller sample of refugees selected from zones that were not included in the study, as part of the training of the research assistants. Minor corrections were made to the questionnaires following this exercise. In addition, upon collection of study data, we tested the instruments for reliability (internal consistency) and found them largely reliable (reported under methods).

Mutiple tests and comparisons increase the risk of getting statistically significant results due to chance (i.e. increased risk for type 1 error). While we attempted to reduce this by using the Bonferroni correction, it has been argued that the method is conservative especially when there are a large number of hypotheses being simultaneously tested and/or testing hypotheses that are highly correlated [56] as may be the case in our study, warranting caution when interpreting our results. Finally, the normality assumption for regressions analysis was not met. However, scholars have recently confirmed using simulations or otherwise that the consequences of violations of the linearity assumption (i.e. biased estimates of coefficients) are critical when samples are small. With large samples (i.e., where the number of observations per variable is > 10), the standard errors of the regression coefficients are unbiased estimates and consequently, the confidence intervals and *p* values [57]. In consideration of this view, we worked with the un-transformed data.

## Conclusion

There is a settlement-inequality (i.e. rural vs. urban) in psychological distress and social support among conflict refugees in Uganda. In order to address psychological distress, Mental Health and Psychosocial Support Services (MHPSS) should focus on strategies to support and strengthen the existing social relations among refugees, in preference to increasing the availability of social networks. Basing on variations in social support as a predictor of distress across different settlements, need-adapted interventions will be more effective than duplication of interventions across settlements.

## Data Availability

The datasets used and/or analysed during the current study are available from the corresponding author on reasonable request.

## References

[1] Dubey S, Biswas P, Ghosh R, Chatterjee S, Dubey MJ, Chatterjee S, Lahiri D, Lavie CJ (2020). Psychosocial impact of COVID-19. Diabetes Metab Syndr.

[2] Public Health Act: Public Health (Control of COVID - 19) (No. 2) Rules, 2020.

[3] Hamza Shuja K, Aqeel M, Jaffar A, Ahmed A (2020). COVID-19 pandemic and impending global mental health implications. Psychiatr Danub.

[4] Josephson A, Kilic T, Michler JD (2021). Socioeconomic impacts of COVID-19 in low-income countries. Nat Hum Behav.

[5] Kansiime MK, Tambo JA, Mugambi I, Bundi M, Kara A, Owuor C (2021). COVID-19 implications on household income and food security in Kenya and Uganda: Findings from a rapid assessment. World Dev.

[6] Kola L (2020). Global mental health and COVID-19. Lancet Psychiatry.

[7] Torales J, O’Higgins M, Castaldelli-Maia JM, Ventriglio A (2020). The outbreak of COVID-19 coronavirus and its impact on global mental health. Int J Soc Psychiatry.

[8] UNDP. The socio-economic impact of COVID-19 in Uganda: short-term and long-term impact on poverty dynamics and SDGs using scenario analysis and system dynamics modelling. 2020. URL file:///Users/gloriaseruwagi/Downloads/Socio-Economic-Impact-COVID-19-Uganda-Brief-1-UNDP-Uganda-April-2020%20(1).pdf

[9] Kumar D, Saghir T, Ali G, Yasin U, Furnaz S, Karim M, Hussain M, Kumari R, Bai R, Kumar H (2021). Psychosocial impact of COVID-19 on healthcare workers at a tertiary care cardiac center of Karachi Pakistan. J Occup Environ Med.

[10] Marazziti D, Pozza A, Di Giuseppe M, Conversano C (2020). The psychosocial impact of COVID-19 pandemic in Italy: a lesson for mental health prevention in the first severely hit European country. Psychol Trauma Theory Res Pract Policy.

[11] Ogrodniczuk JS, Rice SM, Kealy D, Seidler ZE, Delara M, Oliffe JL (2021). Psychosocial impact of the COVID-19 pandemic: a cross-sectional study of online help-seeking Canadian men. Postgrad Med.

[12] Saha K, Torous J, Caine ED, De Choudhury M (2020). Psychosocial effects of the COVID-19 pandemic: large-scale quasi-experimental study on social media. J Med Internet Res.

[13] Saladino V, Algeri D, Auriemma V (2020). The psychological and social impact of Covid-19: new perspectives of well-being. Front Psychol.

[14] Tol WA, Patel V, Tomlinson M, Baingana F, Galappatti A, Panter-Brick C, Silove D, Sondorp E, Wessells M, Van Ommeren M (2011). Research priorities for mental health and psychosocial support in humanitarian settings. PLoS Med.

[15] Tol WA, Barbui C, Galappatti A, Silove D, Betancourt TS, Souza R, Golaz A, Van Ommeren M (2011). Mental health and psychosocial support in humanitarian settings: linking practice and research. The Lancet.

[16] San Lau L, Samari G, Moresky RT, Casey SE, Kachur SP, Roberts LF, Zard M (2020). COVID-19 in humanitarian settings and lessons learned from past epidemics. Nat Med.

[17] Blanchet K, Sistenich V, Ramesh A, Frison S, Warren E, Hossain M, Knight A, Lewis C, Smith J, Woodward A, Dahab M (2013). An evidence review of research on health interventions in humanitarian crises.

[18] Williams RE, Black CL, Kim HY, Andrews EB, Mangel AW, Buda JJ, Cook SF (2006). Determinants of healthcare-seeking behaviour among subjects with irritable bowel syndrome. Aliment Pharmacol Ther.

[19] Barnes DM, Almasy N (2005). Refugees’ perceptions of healthy behaviors. J Immigr Health.

[20] Turrini G, Purgato M, Acarturk C, Anttila M, Au T, Ballette F, Bird M, Carswell K, Churchill R, Cuijpers P, Hall J (2019). Efficacy and acceptability of psychosocial interventions in asylum seekers and refugees: systematic review and meta-analysis. Epidemiol Psychiatric Sci.

[21] Tribe RH, Sendt KV, Tracy DK (2019). A systematic review of psychosocial interventions for adult refugees and asylum seekers. J Ment Health.

[22] Nwadiora E, McAdoo H (1996). Acculturative stress among Amerasian refugees: gender and racial differences. Adolescence.

[23] Bapolisi AM, Song SJ, Kesande C, Rukundo GZ, Ashaba S (2020). Post-traumatic stress disorder, psychiatric comorbidities and associated factors among refugees in Nakivale camp in southwestern Uganda. BMC Psychiatry.

[24] Feyera F, Mihretie G, Bedaso A, Gedle D, Kumera G (2015). Prevalence of depression and associated factors among Somali refugee at melkadida camp, southeast Ethiopia: a cross-sectional study. BMC Psychiatry.

[25] Cohen S, Wills TA (1985). Stress, social support, and the buffering hypothesis. Psychol Bull.

[26] Hobfoll SE (1985). Limitations of social support in the stress process. Social support: theory, research and applications.

[27] Hirsch BJ (1980). Natural support systems and coping with major life changes. Am J Community Psychol.

[28] Roskies E, Lazarus RS. Coping theory and the teaching of coping skills. Behavioral medicine: changing health lifestyles. 1980; 38–69.

[29] Rabin BS, Rabin BC (1999). Stress, immune function, and health: the connection.

[30] Rankin SH, Monahan P (1991). Great expectations: perceived social support in couples experiencing cardiac surgery. Fam Relat.

[31] Wortman CB, Lehman DR, Sarason IG, Sarason BR (1985). Reactions to victims of life crisis: support attempts that fail. Social support: theory, research and applications.

[32] Hobfoll SE, Leiberman Y (1987). Personality and social resources in immediate and continued stress resistance among women. J Pers Soc Psychol.

[33] Leviton LC, Snell E, McGinnis M (2000). Urban issues in health promotion strategies. Am J Public Health.

[34] Geronimus AT (2000). To mitigate, resist, or undo: addressing structural influences on thehealth of urban populations. Am J Public Health.

[35] Vlahov D, Galea S (2002). Urbanization, urbanicity, and health. J Urban Health.

[36] Taylor SE, Sherman DK, Kim HS, Jarcho J, Takagi K, Dunagan MS (2004). Culture and social support: who seeks it and why?. J Pers Soc Psychol.

[37] Godfrey R, Julien M (2005). Urbanisation and health. Clin Med.

[38] UNHCR. Figures at a glance https://www.unhcr.org/figures-at-a-glance.html. Accessed May 2021

[39] Kaiser T (2006). Between a camp and a hard place: rights, livelihood and experiences of the local settlement system for long-term refugees in Uganda. J Mod African Stud.

[40] Ministry of Health. COVID-19 response info hub. 2020. https://covid19.gou.go.ug/timeline.html.

[41] Nakalembe JS. Covid 19 interventions verses a woman in Uganda [Internet]. Stop Gender Based Violence [cited 2021 Apr 20]. 2020. https://www.cehurd.org/covid-19-interventions-verses-a-woman-in-uganda/

[42] Gato J. The Effects of COVID-19 Lockdown on Urban Refugees in Kampala. [Internet]. https://blog.fluchtforschung.net/covid-19-lockdown-on-urban-refugees-in-kampala/. 2020.

[43] Bukuluki P, Mwenyango H, Katongole SP, Sidhva D, Palattiyil G (2020). The socio-economic and psychosocial impact of Covid-19 pandemic on urban refugees in Uganda. Social Sci Humanit Open.

[44] Guadagno L (2020). Migrants and the COVID-19 pandemic: an initial analysis.

[45] Truelove S, Abrahim O, Altare C, Lauer SA, Grantz KH, Azman AS, Spiegel P (2020). The potential impact of COVID-19 in refugee camps in Bangladesh and beyond: a modeling study. PLoS Med.

[46] Tsamakis K, Tsiptsios D, Ouranidis A, Mueller C, Schizas D, Terniotis C, Nikolakakis N, Tyros G, Kympouropoulos S, Lazaris A, Spandidos DA, Smyrnis N, Rizos E (2021). COVID-19 and its consequences on mental health. Exp Ther Med.

[47] Kessler RC, Andrews G, Colpe LJ, Hiripi E, Mroczek DK, Normand SL, Walters EE, Zaslavsky AM (2002). Short screening scales to monitor population prevalences and trends in non-specific psychological distress. Psychol Med.

[48] Henderson S, Duncan-Jones P, Byrne DG, Scott R (1980). Measuring social relationships the interview schedule for social interaction. Psychol Med.

[49] Norbeck JS, Lindsey AM, Carrieri VL (1981). The development of an instrument to measure social support. Nurs Res.

[50] Kaplan BH, Cassel JC, Gore S (1977). Social support and health. Med Care.

[51] Berkman LF, Syme SL (1979). Social networks, host resistance, and mortality: a nine-year follow-up study of Alameda County residents. Am J Epidemiol.

[52] Ahimbisibwe F. Uganda and the refugee problem: Challenges and opportunities. Institute of Development Policy and Management (IOB), University of Antwerp, Working Paper No. 2018 May.

[53] Monteith W, Lwasa S (2017). The participation of urban displaced populations in (in) formal markets: contrasting experiences in Kampala, Uganda. Environ Urban.

[54] Peltzer K (1999). Trauma and mental health problems of Sudanese refugees in Uganda. Cent Afr J Med.

[55] Hovil L (2007). Self-settled refugees in Uganda: an alternative approach to displacement?. J Refug Stud.

[56] Schmidt AF, Finan C (2018). Linear regression and the normality assumption. J Clin Epidemiol.

[57] Chen SY, Feng Z, Yi X (2017). A general introduction to adjustment for multiple comparisons. J Thorac Dis.

